# Perspectives of Patients About Artificial Intelligence in Health Care

**DOI:** 10.1001/jamanetworkopen.2022.10309

**Published:** 2022-05-04

**Authors:** Dhruv Khullar, Lawrence P. Casalino, Yuting Qian, Yuan Lu, Harlan M. Krumholz, Sanjay Aneja

**Affiliations:** 1 Division of Health Policy and Economics, Department of Population Health Sciences, Weill Cornell Medical College, New York, New York; 2 Division of General Internal Medicine, Department of Medicine, Weill Cornell Medical College, New York, New York; 3 Center for Outcomes Research and Evaluation, Yale School of Medicine, New Haven, Connecticut; 4 Department of Therapeutic Radiology, Yale School of Medicine, New Haven, Connecticut

## Abstract

This survey study describes the results of a nationally representative survey on artificial intelligence use in health care delivery.

## Introduction

Applications of artificial intelligence (AI) in health care have increased in the past decade,^1^ but little is known about how patients view these applications and whether they have concerns.^2^ We conducted a nationally representative survey to understand public perceptions of the use of AI in diagnosis and treatment.

## Methods

The survey was administered between December 3, 2019, and December 18, 2019, by an independent research firm using a hybrid probability-based, nationally representative online panel, which was weighted to correct for biases in sampling and nonresponse across demographic groups^3^ (eAppendix 1 in the Supplement). This survey study was deemed exempt from review by the institutional review boards at Yale University and Weill Cornell because identities were kept confidential. We followed the (AAPOR) reporting guideline.

Adjusted response rate (54%) was calculated using the RR3 formula of the American Association for Public Opinion Research.^2,4^ χ^2^ tests were used for statistical analysis across demographic characteristics, and significance was set at 2-sided *P* < .05.

## Results

A total of 926 respondents (471 women [50.9%], 455 men [49.1%]) completed the survey (eAppendix 2 in the Supplement). Most patients believed that AI would make health care much better (10.9%) or somewhat better (44.5%), whereas some believed AI would make health care somewhat worse (4.3%) or much worse (1.9%); 19% indicated they did not know (Table 1).

**Table 1.** Public Views About Artificial Intelligence in Health Care

Response	Respondents, No. (%)						
	Total (n = 926)	Age		Race and ethnicity^a^		Answer to question 1^b^	
		18-49 y (n = 533)	≥50 y (n = 392)	White (n = 551)	Other (n = 350)^c^	Don’t know (n = 176)	Other answer (n = 750)
**Overall, in the next 5 y, do you think AI will make health care in the United States?**							
Much better	101 (10.9)	60 (11.3)	41 (10.5)	63 (11.4)	35 (10.0)	NA	NA
Somewhat better	412 (44.5)	239 (44.8)	173 (44.1)	245 (44.5)	161 (46.0)	NA	NA
Minimal change	179 (19.3)	105 (19.7)	73 (18.6)	110 (20.0)	61 (17.4)	NA	NA
Somewhat worse	40 (4.3)	22 (4.1)	18 (4.6)	26 (4.7)	12 (3.4)	NA	NA
Much worse	18 (1.9)	10 (1.9)	8 (2.0)	9 (1.6)	7 (2.0)	NA	NA
Don’t know	176 (19.0)	97 (18.2)	79 (20.2)	98 (17.8)	74 (21.1)	NA	NA
**How important do you think it is that you are told when an AI program has played a big role in your diagnosis or treatment?**							
Not important	39 (4.2)	25 (4.7)	14 (3.6)	24 (4.4)	13 (3.7)	7 (4.0)	32 (4.3)
Somewhat important	276 (29.8)	167 (31.3)	109 (27.8)	159 (28.9)	110 (31.4)	41 (23.3)	235 (31.3)
Very important	611 (66.0)	341 (64.0)	269 (68.6)	368 (66.8)	227 (64.9)	128 (72.7)	483 (64.4)
**How important do you think it is that you are told when an AI program has played a small role in your diagnosis or treatment?**							
Not important	125 (13.5)	83 (15.6)	42 (10.7)	77 (14.0)	45 (12.9)	10 (5.7)	115 (15.3)
Somewhat important	379 (40.9)	239 (44.8)	140 (35.7)	221 (40.1)	149 (42.6)	61 (34.7)	318 (42.4)
Very important	422 (45.6)	211 (39.6)	210 (53.6)	253 (45.9)	156 (44.6)	105 (59.7)	317 (42.3)
**How comfortable would you be receiving a diagnosis from a computer program that made the right diagnosis 90% of the time but could not explain why it made the diagnosis?**							
Very uncomfortable	287 (31.0)	168 (31.5)	118 (30.1)	167 (30.3)	110 (31.4)	56 (31.8)	231 (30.8)
Somewhat uncomfortable	375 (40.5)	207 (38.8)	168 (42.9)	231 (41.9)	137 (39.1)	72 (40.9)	303 (40.4)
Somewhat comfortable	204 (22.0)	124 (23.3)	80 (20.4)	120 (21.8)	78 (22.3)	39 (22.2)	165 (22.0)
Very comfortable	60 (6.5)	34 (6.4)	26 (6.6)	33 (6.0)	25 (7.1)	9 (5.1)	51 (6.8)
**How comfortable would you be receiving a diagnosis from a computer program that made the right diagnosis 98% of the time but could not explain why it made the diagnosis?**							
Very uncomfortable	188 (20.3)	113 (21.2)	74 (18.9)	104 (18.9)	75 (21.4)	47 (26.7)	141 (18.8)
Somewhat uncomfortable	349 (37.7)	201 (37.7)	148 (37.8)	196 (35.6)	144 (41.1)	68 (38.6)	281 (37.5)
Somewhat comfortable	297 (32.1)	170 (31.9)	127 (32.4)	190 (34.5)	103 (29.4)	49 (27.8)	248 (33.1)
Very comfortable	92 (9.9)	49 (9.2)	43 (11.0)	61 (11.1)	28 (8.0)	12 (6.8)	80 (10.7)

Abbreviations: AI, artificial intelligence; NA, not applicable.

^a^ Race and ethnicity were self-identified.

^b^ Question 1: Overall, in the next 5 years, do you think AI will make health care in the United States?

^c^ Other (than White Hispanic and White non-Hispanic) race and ethnicity included Asian, Chinese, or Japanese; Black Hispanic; Black non-Hispanic; unspecified Hispanic; Native American, American Indian, or Alaska Native; Native Hawaiian and other Pacific Islander; other race; mixed race; and refused to answer.

Regarding being informed if AI played a big role in their diagnosis or treatment, 66% of respondents deemed it very important and 29.8% stated it was somewhat important. Thirty-one percent of respondents reported being very uncomfortable and 40.5% were somewhat uncomfortable with receiving a diagnosis from an AI algorithm that was accurate 90% of the time but incapable of explaining its rationale. Responses were similar by age and race and ethnicity. Compared with respondents who shared their views about the potential implications of AI for health care, more respondents who answered with “don’t know” deemed it very important to be told when AI played a small role in their diagnosis or treatment (59.7% vs 42.3%) and were very uncomfortable with receiving an AI diagnosis that was accurate 98% of the time but could not be explained (26.7% vs 18.8%) (Table 1).

Comfort with AI varied by clinical application (Table 2). For example, 12.3% of respondents were very comfortable and 42.7% were somewhat comfortable with AI reading chest radiographs, but only 6.0% were very comfortable and 25.2% were somewhat comfortable about AI making cancer diagnoses. Most respondents were very concerned or somewhat concerned about AI’s unintended consequences, including misdiagnosis (91.5%), privacy breaches (70.8%), less time with clinicians (69.6%), and higher health care costs (68.4%). A higher proportion of respondents who self-identified as being members of racial and ethnic minority groups indicated being very concerned about these issues, compared with White respondents.

**Table 2.** Public Comfort and Concerns With Artificial Intelligence in Health Care

	Respondents, No. (%)				
	Total (n = 926)	Age 18-49 y (n = 533)	Age ≥50 y (n = 392)	White race^a^ (n = 551)^b^	Other race and ethnicity (n = 350)^a^^,^^c^
**How comfortable you would feel with AI doing some of the things your doctor usually does for each of the following**					
Reading your chest x-ray					
Very uncomfortable	159 (17.2)	89 (16.7)	70 (17.9)	89 (16.2)	64 (18.3)
Somewhat uncomfortable	258 (27.9)	142 (26.6)	115 (29.3)	162 (29.4)	86 (24.6)
Somewhat comfortable	395 (42.7)	223 (41.8)	172 (43.9)	233 (42.3)	154 (44.0)
Very comfortable	114 (12.3)	79 (14.8)	35 (8.9)	67 (12.2)	46 (13.1)
Making the diagnosis of pneumonia					
Very uncomfortable	180 (19.4)	95 (17.8)	84 (21.4)	103 (18.7)	69 (19.7)
Somewhat uncomfortable	302 (32.6)	178 (33.4)	124 (31.6)	164 (29.8)	130 (37.1)
Somewhat comfortable	357 (38.6)	205 (38.5)	152 (38.8)	230 (41.7)	119 (34.0)
Very comfortable	87 (9.4)	55 (10.3)	32 (8.2)	54 (9.8)	32 (9.1)
Telling you that you have pneumonia					
Very uncomfortable	257 (27.8)	148 (27.8)	108 (27.6)	138 (25.0)	110 (31.4)
Somewhat uncomfortable	325 (35.1)	178 (33.4)	147 (37.5)	203 (36.8)	113 (32.3)
Somewhat comfortable	258 (27.9)	143 (26.8)	115 (29.3)	152 (27.6)	101 (28.9)
Very comfortable	85 (9.2)	64 (12.0)	21 (5.4)	57 (10.3)	26 (7.4)
Recommending the type of antibiotics you get					
Very uncomfortable	192 (20.7)	103 (19.3)	89 (22.7)	107 (19.4)	76 (21.7)
Somewhat uncomfortable	248 (26.8)	130 (24.4)	117 (29.8)	144 (26.1)	97 (27.7)
Somewhat comfortable	359 (38.8)	215 (40.3)	144 (36.7)	217 (39.4)	134 (38.3)
Very comfortable	127 (13.7)	85 (15.9)	42 (10.7)	83 (15.1)	43 (12.3)
Making the diagnosis of cancer					
Very uncomfortable	396 (42.8)	216 (40.5)	179 (45.7)	225 (40.8)	160 (45.7)
Somewhat uncomfortable	241 (26.0)	138 (25.9)	103 (26.3)	154 (27.9)	80 (22.9)
Somewhat comfortable	233 (25.2)	138 (25.9)	95 (24.2)	139 (25.2)	88 (25.1)
Very comfortable	56 (6.0)	41 (7.7)	15 (3.8)	33 (6.0)	22 (6.3)
Telling you that you have cancer					
Very uncomfortable	533 (57.6)	297 (55.7)	235 (59.9)	322 (58.4)	200 (57.1)
Somewhat uncomfortable	224 (24.2)	121 (22.7)	103 (26.3)	141 (25.6)	74 (21.1)
Somewhat comfortable	120 (13.0)	78 (14.6)	42 (10.7)	59 (10.7)	56 (16.0)
Very comfortable	49 (5.3)	37 (6.9)	12 (3.1)	29 (5.3)	20 (5.7)
**How concerned you are about the use of AI in medicine for each of the following**					
My health information will not be kept confidential					
Not concerned	270 (29.2)	163 (30.6)	107 (27.3)	174 (31.6)	91 (26.0)
Somewhat concerned	359 (38.8)	204 (38.3)	155 (39.5)	227 (41.2)	124 (35.4)
Very concerned	297 (32.1)	166 (31.1)	130 (33.2)	150 (27.2)	135 (38.6)
The AI will make the wrong diagnosis					
Not concerned	79 (8.5)	48 (9.0)	31 (7.9)	51 (9.3)	25 (7.1)
Somewhat concerned	477 (51.5)	267 (50.1)	209 (53.3)	302 (54.8)	161 (46.0)
Very concerned	370 (40.0)	218 (40.9)	152 (38.8)	198 (35.9)	164 (46.9)
AI will mean I spend less time with my doctor					
Not concerned	280 (30.2)	178 (33.4)	102 (26.0)	171 (31.0)	104 (29.7)
Somewhat concerned	356 (38.4)	184 (34.5)	172 (43.9)	219 (39.7)	126 (36.0)
Very concerned	289 (31.2)	170 (31.9)	118 (30.1)	161 (29.2)	119 (34.0)
AI will increase my health care costs					
Not concerned	293 (31.6)	181 (34.0)	112 (28.6)	195 (35.4)	90 (25.7)
Somewhat concerned	298 (32.2)	155 (29.1)	142 (36.2)	189 (34.3)	101 (28.9)
Very concerned	335 (36.2)	197 (37.0)	138 (35.2)	167 (30.3)	159 (45.4)

Abbreviation: AI, artificial intelligence.

^a^ Race and ethnicity were self-identified.

^c^ Other (than White Hispanic and White non-Hispanic) race and ethnicity included Asian, Chinese, or Japanese; Black Hispanic; Black non-Hispanic; unspecified Hispanic; Native American, American Indian, or Alaska Native; Native Hawaiian and other Pacific Islander; other race; mixed race; and refused to answer.

## Discussion

Most respondents had positive views about AI’s ability to improve care but had concerns about its potential for misdiagnosis, privacy breaches, reducing time with clinicians, and increasing costs, with racial and ethnic minority groups expressing greater concern. Respondents were more comfortable with AI in specific clinical settings, and most wanted to know when AI was used in their care.

One limitation of this study was it involved a panel that had agreed to participate in surveys, which may limit generalizability. In addition, compared with nonrespondents, respondents were younger, but no significant differences by sex or race and ethnicity were found.

Clinicians, policy makers, and developers should be aware of patients’ views regarding AI. Patients may benefit from education on how AI is being incorporated into care and the extent to which clinicians rely on AI to assist with decision-making. Future work should examine how views evolve as patients become more familiar with AI.

## Notes

Supplement. **eAppendix 1.** Supplemental Survey Methods and Pilot Testing **eAppendix 2.** Patients’ Views of AI in Health Care Survey
