# Supplementary material for: Perspectives of Patients About Artificial Intelligence in Health Care
Source: JAMA Netw Open. 2022 May 4;5(5):e2210309. doi: 10.1001/jamanetworkopen.2022.10309 (PMC9069257; doi:10.1001/jamanetworkopen.2022.10309)
Supplement: Supplement. — eAppendix 1. Supplemental Survey Methods and Pilot Testing eAppendix 2. Patients’ Views of AI in Health Care Survey [file jamanetwopen-e2210309-s001.pdf]

Supplementary Online Content

Khullar D, Casalino LP, Qian Y, Lu Y, Krumholz HM, Aneja S. Perspectives of patients about artificial intelligence in health care. *JAMA Netw Open*. 2022;5(5):e2210309. doi:10.1001/jamanetworkopen.2022.10309

eAppendix 1. Supplemental Survey Methods and Pilot Testing

eAppendix 2. Patients' Views of AI in Health Care Survey

This supplementary material has been provided by the authors to give readers additional information about their work.

© 2022 Khullar D et al. *JAMA Network Open.*

eAppendix 1. Supplemental Survey Methods and Pilot Testing

We conducted surveys of the general public which was administered by SSRS, an independent research firm.[1] Each survey instrument was developed through literature review and expert input from physicians and SSRS. Surveys were pilot tested with 10 members of the general public for clarity and length, and revised accordingly. The public survey used a hybrid probability-based online panel and a dual-frame random-digit-dial telephone sample. Online participants were drawn from the SSRS Opinion Panel, which is representative of the online adult U.S. population.[2] The sample was supplemented with the SSRS Omnibus sample, a national, weekly telephone survey[3] conducted in English and Spanish.

References

1. SSRS Full-service Survey and Market Research Firm. https://ssrs.com/. Accessed December 16, 2021.

2. Opinion Panel. SSRS. https://ssrs.com/opinion-panel/. Accessed October 26, 2021.

3. SSRS Omnibus Survey. https://ssrs.com/ssrs-omnibus-survey/. Accessed October 26, 2021.

© 2022 Khullar D et al. *JAMA Network Open.*

eAppendix 2. Patients' Views of AI in Health Care Survey

Job #S1119
September 2019

Patient AI in Healthcare Survey

Self (Web) Administration

(ASK ALL)
(IF WEB SHOW: Welcome and thank you for participating in our short survey about healthcare!)

Q1. Artificial intelligence, known as "AI", is now being used in some parts of health care. AI usually refers to computer programs that can copy human intelligence and learn automatically. AI is being tested to see if computer programs can do things like read chest x-rays, help make diagnoses (DIE-AG-NO-SEES), and figure out when patients will get sick.

Which, if any, of these technologies do you use at least once a month?

(IF WEB SHOW)

1 Smart phone
2 Online shopping websites
3 Mobile banking
4 Website to view my electronic medical records
0 None of these
X Blank

(ASK ALL)
Q2. How often have you seen a doctor for medical care in the past year?

(IF WEB SHOW)

1 I have not seen a doctor for medical care in the last year
2 Once or twice
3 Three to five times
4 More than five times
X Blank

(ASK ALL)
Q3. Would you say that, in general, your overall health is:

5 Excellent
4 Very good
3 Good

© 2022 Khullar D et al. *JAMA Network Open.*

2 Fair
1 Poor
8 Don't know
9 Refused
X Blank

(ASK ALL)

Q4. How much do you know about Artificial Intelligence (AI) and how it could change medicine?

(IF WEB SHOW)

4 I know quite a lot
3 I know a fair amount
2 I know a little bit
1 I know almost nothing

(ASK ALL)

Q5. Overall, in the next 5 years, do you think AI will make health care in the United States:

5 Much better
4 Somewhat better
3 Minimal change
2 Somewhat worse
1 Much worse
8 Don't know
9 Refused
X Blank

(ASK ALL)

Q6. [PN: IF WEB DISPLAY: For each of the following, how comfortable would you feel with AI doing some of the things your doctor usually does.]

4 Very comfortable
3 Somewhat comfortable
2 Somewhat uncomfortable
1 Very uncomfortable
8 Don't know
9 Refused
X Blank

(IF WEB SHOW AS A GRID WITH THE RESPONSE OPTIONS 4-1 ACROSS THE TOP AND STUBS AS ROWS)

a. AI, instead of your doctor, reading your chest x-ray?
b. AI, instead of your doctor, making the diagnosis of pneumonia?
c. AI, instead of your doctor, telling you that you have pneumonia?
d. AI, instead of your doctor, recommending the type of antibiotics you get?
e. AI, instead of your doctor, making the diagnosis of cancer?
f. AI, instead of your doctor, telling you that you have cancer?

© 2022 Khullar D et al. *JAMA Network Open.*

(ASK ALL)

Q7. [PN: IF WEB DISPLAY: Please rate how concerned you are about the use of AI in medicine for each of the following.]

3 Very concerned
2 Somewhat concerned
1 Not concerned
8 Don't know
9 Refused
X Blank

(IF WEB SHOW AS A GRID WITH THE RESPONSE OPTIONS 3-1 ACROSS THE TOP AND STUBS AS ROWS)

a. That my health information will not be kept confidential
b. That the AI will make the wrong diagnosis
c. That AI will mean I spend less time with my doctor
d. That AI will increase my health care costs
e. Anything else you might be concerned about? (PLEASE SPECIFY______________)

(ASK ALL)

Q8. [PN IF WEB DISPLAY: Next, how comfortable would you feel sharing private information (for example, how many sexual partners you have had, whether you have taken drugs, or whether you have had a miscarriage). For each of the following, how comfortable would you be with…?]

1 Very uncomfortable
2 Somewhat uncomfortable
3 Somewhat comfortable
4 Very comfortable
8 Don't know
9 Refused
X Blank

(IF WEB SHOW AS A GRID WITH THE RESPONSE OPTIONS 1-4 ACROSS THE TOP AND STUBS AS ROWS)

a. A human doctor if you believe it will be kept secret
b. An AI chatbot if you believe it will be kept secret

(ASK ALL)

Q9. How comfortable would you feel anonymously sharing your health information to help improve AI programs that treat disease?

1 Very uncomfortable
2 Somewhat uncomfortable
3 Somewhat comfortable
4 Very comfortable

© 2022 Khullar D et al. *JAMA Network Open.*

8 Don’t know
9 Refused
X Blank

(ASK ALL)

Q10. How important do you think it is that you are told when an AI program has played a *big* role in your diagnosis or treatment?

1 Not important
2 Somewhat important
3 Very important
8 Don’t know
9 Refused
X Blank

(ASK ALL)

Q11. How important do you think it is that you are told when an AI program has played a *small* role in your diagnosis or treatment?

1 Not important
2 Somewhat important
3 Very important
8 Don’t know
9 Refused
X Blank

(ASK ALL)

Q12. Imagine that you have been diagnosed with atrial fibrillation, an abnormal heart rhythm that increases your chances of having a stroke. In the past, your doctor would have decided whether you need to start a blood thinning medicine based on things like your age and other medical problems.

Your doctor now has a computer program that uses many other factors. This computer program says you should start a blood thinner. How important is it that your doctor tells you that the computer program helped make this decision?

1 Not important
2 Somewhat important
3 Very important
8 Don’t know
9 Refused
X Blank

(ASK ALL)

Q13. How comfortable would you be receiving a diagnosis from a computer program that made the right diagnosis **90%** of the time but could not explain why it made the diagnosis?

© 2022 Khullar D et al. *JAMA Network Open.*

1 Very uncomfortable
2 Somewhat uncomfortable
3 Somewhat comfortable
4 Very comfortable
8 Don't know
9 Refused
X Blank

(ASK ALL)

Q14. How comfortable would you be receiving a diagnosis from a computer program that made the right diagnosis **98%** of the time but could not explain why it made the diagnosis?

1 Very uncomfortable
2 Somewhat uncomfortable
3 Somewhat comfortable
4 Very comfortable
8 Don't know
9 Refused
X Blank

(ASK ALL)

Q15. Imagine that you have some symptoms that have been bothering you for a while, such as difficulty sleeping, rashes, and joint pain. You visit a doctor who runs some tests and he says he does NOT think you have Lupus. He also puts your symptoms into a computer program that can make the right diagnosis about 80% of the time, but can't say why it chose the diagnoses. It says you DO have Lupus. How does the computer program affect your view?

1 It would not affect my trust of the doctor's assessment.
2 It would make me question the doctor's assessment.
3 I do not know if it would change my view of the doctor's assessment.
8 Don't know
9 Refused
X Blank

(ASK ALL)

Q16. Imagine that your doctor and a computer program work together to treat you and a medical error occurs. An example of a medical error is getting a diagnosis that was wrong, or a treatment that was not needed. Who is responsible?

(IF WEB SHOW) (Please select all that apply.)
1 The doctor who made the decision
2 The company that made the computer program

3 The hospital that bought the computer program
4 The government agency that approved the computer program
7 Someone else (Please specify:____)
0 No one
8 Don’t know
9 Refused
X Blank

(ASK ALL)

Q17. Imagine that you have a mole that might be cancer. You have a biopsy done. Your doctor uses a computer program that says the mole might be cancer so you have a procedure to remove it. The procedure leads to bad complications. After another doctor looks at the mole, it turns out it was NOT cancer. Who, if anyone, is to blame?

(IF WEB SHOW) (Please select all that apply.)
1 The doctor who made the decision
2 The company that made the computer program
3 The hospital that bought the computer program
4 The government agency that approved the computer program
7 Someone else (Please specify:____)
0 No one
8 Don’t know
9 Refused
X Blank

(ASK ALL)

Q18. Imagine that your hospital recently started using a computer program to help diagnose some medical problems. Who do you think has checked to make sure the computer program is safe before it is rolled out?

(IF WEB SHOW) (Please select all that apply.)
1 The doctor who made the decision
2 The company that made the computer program
3 The hospital that bought the computer program
4 The government agency that approved the computer program
7 Someone else (Please specify:____)
0 No one
8 Don’t know
9 Refused
X Blank

That completes the survey. Have a great day!

© 2022 Khullar D et al. *JAMA Network Open.*
